# PA12 Powder Recycled from SLS for FDM

**DOI:** 10.3390/polym11040727

**Published:** 2019-04-22

**Authors:** Li Feng, Yan Wang, Qinya Wei

**Affiliations:** School of Material Science and Engineering, Wuhan Institute of Technology, Wuhan 430074, Hubei, China; fenglifl0512@163.com (L.F.); 13477364887@163.com (Q.W.)

**Keywords:** recycled PA12 powder, filament, FDM, SLS

## Abstract

In this study, Polyamide 12 (PA12) powder recycled after selective laser sintering (SLS) was made into filaments for fused deposition modelling (FDM). Compared with fresh PA12, the melt flow rate (MFR) of the recycled PA12 powder decreased by 77%, but the mechanical properties were only slightly reduced. In FDM, the printing speed and building orientation were changed, and the performance of the printed parts was tested. If the printing speed is too fast or too slow, the mechanical properties of the parts will be affected, and there is an optimal speed range. The tensile strength, flexural modulus, and impact strength of a printed test sample made from recycled powder reached 95%, 85%, and 87% of an *x*-direction test sample made from fresh PA12, respectively. For test samples printed from different orientations, the mechanical properties of the test samples printed in the *x*-direction were the best, while the crystallization performance was the opposite. Scanning electron microscope (SEM) images show that the printed test sample had good compactness and mechanical properties, and the delamination phenomenon was basically not observed.

## 1. Introduction

Three-dimensional printing technology (also known as additive manufacturing (AM) technology) is based on 3D model data, and it uses a method of layer-by-layer accumulation of materials to manufacture solid parts, in contrast to traditional removal manufacturing (cutting processing). The process can quickly manufacture complex parts of any shape and structure in a short cycle and achieve individualized manufacturing results [1,2]. Fused deposition modelling (FDM) and selective laser sintering (SLS) are two of the most widely used technologies [3,4,5].

Fused deposition modelling has become an important branch of 3D printing due to its advantages of safety, environmental protection, wide range of materials, high integration, and low cost [6]. Fused deposition modelling technoloy controls a nozzle via computer according to geometric information (3D graphics) determined by a CAD model. The material is melted into liquid through the extrusion head of the heater. The molten thermoplastic filament is extruded through the nozzle, and the extrusion head moves accurately along the contour of each section of the part. The semi-flowing thermoplastic material is extruded, and the deposition is solidified into a precise thin layer of the actual component, overlaid on the built parts and solidified rapidly within 1/10 s to form a layer of material. After that, the printing platform moves downwards in the axial direction by one layer, and then the next layer of material is constructed. A solid model or part is stacked layer by layer from bottom to top [7,8].

In the SLS process, a CO_2_ laser is used as the energy source, and a computer is used to control the laser beam and scan the powder material at a certain speed and energy density according to pre-set two-dimensional data, stacking layer upon layer, finally forming a molded part [9,10,11,12]. Polyamide 12 has high crystallinity, high strength, a low melting point, and low water absorption and forming shrinkage, so it exhibits good comprehensive performance, including heat resistance, wear resistance, flame retardancy, easy processing, etc., which makes it the most commonly used material for the rapid manufacturing of functional components in the SLS process, accounting for more than 95% of the SLS polymer powder material market. At present, more research is on the PA12 composite powder. Zhu et al. [13] used a new method to prepare a high-performance SLS carbon fiber/polyamide 12/epoxy (CF/PA12/EP) ternary composite. Li et al. [14] prepared a glass fiber/phenolic resin/epoxy resin (GF/PF/EP) three-phase electrical insulating composite material for SLS, and Yan et al. [15] prepared a carbon fiber/polyamide-12 (CF/PA) composite powder. In SLS, the average total volume of manufactured parts is relatively small, between 5% and 15% of the volume of the manufactured article, which means that 85% to 95% of the material is not sintered and can theoretically be recycled [16].

To prevent sintered parts from warping and deforming during the laser sintering process, the powder layer is usually pre-heated, and the heating temperature is very close to the material melting temperature, which causes physical and chemical changes in the powder material. Chen et al. [17] studied systematical mechanisms of PA-12 aging and its micro-structural evolution during SLS. It was found that the effect of solid-state polycondensation reduces the crystallinity of the powder by ∼6% after three recycling. Pham et al. [16] found that in SLS, the PA12 powder undergoes molecular chain entanglement to form spherulites. The radius of the spherulites is a positive function of time and temperature. The higher the temperature and the longer the powder is exposed to high temperatures, the more likely that the molecular chains become larger, which eventually leads to an increase in molecular weight, a decrease in fluidity, and ultimately the deterioration of the powder’s mechanical and thermal properties. Therefore, the re-use of non-sintered powder is limited and must be updated with a large amount of fresh materials; otherwise, the quality of the manufactured parts will change, the shrinkage rate will be higher, and the surface roughness will be greater, a phenomenon referred to as “orange peel”. To some extent, old materials should be completely discarded to avoid significant deterioration in part quality and waste of fresh materials. However, SLS powder is a relatively expensive material, and its cost has formed a large part of the total cost of SLS manufacturing. Abandonment not only wastes resources but also pollutes the environment. Polyamide 12 powder designed for SLS costs around $150/kg, while the cost of PA12 pellets for conventional plastics processing is below $3/kg. The cost of PA12 filament for FDM is approximately $100/kg. Hence, turning SLS powder residue into polymer filaments for FDM can be one of the solutions to its successful reuse [18]. In SLS molding, the movement of polymer powder mainly occurs via viscous flow [19]. The melt viscosity of a polymer powder is an important parameter that determines whether it can be sintered. Polyamide 12 condenses during sintering, and the molecular weight increases, resulting in poor melt flowability, so it cannot be sintered again. However, FDM has a wider processing range, and the powder at this time is still available for FDM. This will help connect two separate additive manufacturing (AM) processes (i.e., SLS and FDM) to make AM more cost-effective, economical, and environmentally friendly.

At present, research on FDM-forming materials has mainly concentrated on polylactic acid (PLA) and acrylonitrile butadiene Styrene copolymers (ABS) [20]. These two materials perform poorly in terms of high temperature and corrosion resistance, which restricts their application in industry. Nylon has these advantages, but it is mainly used in SLS. There is little research on the use of nylon for FDM. Rahim et al. [21] compounded PA12 powder with zirconia and hydroxyapatite for filament fabrication and final FDM printing. Compared with molded test samples, printed test samples made by composite filaments have good mechanical properties. Composite materials can be used for non-loaded bio-implant materials. Hao Li et al. [14] used PA12 pellets and ABS pellets to produce filaments and printed test samples. Compared with amorphous ABS, FDM parts printed by semi-crystalline PA12 have better adhesive quality and higher tensile strength. In another study, Singh et al. [22] recycled discarded PA6 wire and prepared a filament with Al and Al_2_O_3_ for FDM printing, which reduced the operating cost of the RepRap system.

In this study, waste SLS powder was collected and processed in a single-screw extruder to prepare filaments for FDM, while the same experiment was performed with PA12 pellets. Test samples were printed with the prepared filaments and then tested and characterized to determine their properties and suitability for FDM.

## 2. Experimental Procedure

### 2.1. Materials and Processing

Recycled PA12 powder used in this test was supplied by Degussa GmbH, Germany, which was dried at 60 °C for three hours by a conventional air oven and extruded granulated by a SHJ-36 twin-screw extruder (Nanjing GIANT Machinery Co., Ltd., Nanjing, China). The twin-screw extruder was pre-heated, and the temperature was set to 225 °C in the first zone, 240 °C in the second zone, 255 °C in the third zone, 260 °C in the fourth zone, 260 °C in the fifth zone, and 240 °C in the head. The PA12 recycled powder was weighed, poured into a twin-screw extruder in a batch, melt-blended, and subjected to extrusion granulation. The obtained pellets were cooled for injection molding.

### 2.2. Injection Moulding

The purchased fresh PA12 pellets (Degussa GmbH, Essen, Germany) and the pellets obtained from the recycled powder, as well as the recycled powder, were separately molded into a test sample for testing tensile (ISO 527-2:1993), flexural (ISO 178:2001), and impact properties (ISO 180:2000). The barrel temperature of the injection molding machine was set to 230 °C, the injection time was 6 s, the clamping time was 6 s, and the cooling time was 6 s. Ten straight test samples and 5 dumbbell-shaped test samples were made in each group. The molded test samples were sealed in bags, and the mechanical properties and thermal properties were tested after 24 hours of equilibration.

### 2.3. Melt Mass Flow Rate (MFR)

A small amount of purchased PA 12 pellets, pellets obtained by recycled powder, and recycled powder were dried in an oven at 80 °C for 2 hours. MFI was done as ISO 1133:1997 by an extrusion plastometer (model SRZ-400D, Changchun city intelligent instrument equipment Co., Ltd., Changchun, China). The experiment was set to cut the sample every 6 s. The test conditions were a temperature of 230 °C and a load of 2.16 kg, and the obtained samples were weighed separately. The average value was used to calculate the MFR, and the remaining material was sealed for use.

### 2.4. Filament Preparation and FDM Printing

The purchased PA12 pellets were weighed and then dried in an oven at 80 °C for 5 hours. The 3D consumable extruder (Dongguan Songhu Plastic Machinery Corp., Dongguan, China) was pre-heated, and the temperature was set to 210 °C in the first temperature zone, 240 °C in the second temperature zone, 220 °C in the third temperature zone, and 50 °C in the fourth temperature zone. The dried pellets were then extruded and drawn in batches by a 3D consumable extruder. The speed of the machine and the tractor was adjusted, the diameter of the filament was controlled at approximately 1.75 mm, and the prepared filament was rolled. In the same manner, the pellets obtained from the recycled PA12 powder were dried, and the pellets were extruded into a PA12 filament with a diameter of approximately 1.75 mm by a 3D consumable extruder.

**Figure 1:**
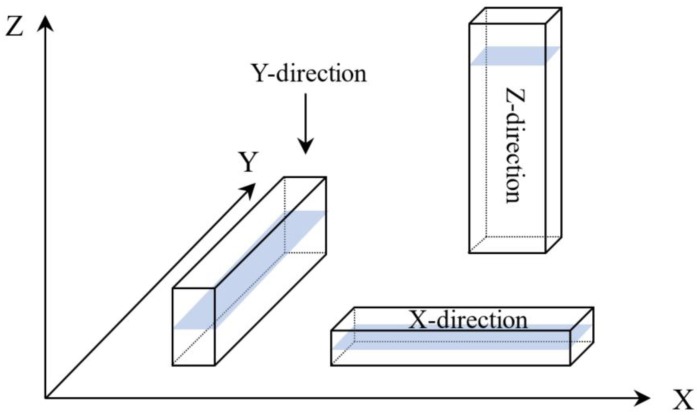
Build orientation.

The two types of PA12 filaments were printed into test samples by an FDM printer (MAGIC-L, Dongguan Yimai Intelligent Technology Co., Ltd., Dongguan, China) in the *x*-direction, *y*-direction, and *z*-direction, the specific orientation of printing is shown in Figure 1. The printer parameters were set to a layer height of 0.1 mm, a shell thickness of 1.2 mm, a bottom thickness of 1.2 mm, a bottom layer printing speed of 20 mm/s, a printing speed of 40 mm/s, a printing temperature of 235 °C, a hot bed temperature of 110 °C, and a packing density of 100%. The print speed parameter was set as a variable, and its print performance was tested.

### 2.5. Mechanical Performance Test

According to ISO 178:2001, the molded test sample and the printed test sample were prepared as notch samples using a notch sample making machine, and then the flexural properties were tested with an electronic tensile testing machine (model TCS-2000, GOTECH TESTING MACHINES INC., Taichung, Taiwan) at a flexural test rate of 2 mm/min. The tensile properties were tested as ISO 527-1:1993 by an electronic tensile testing machine (model TCS-2000) at a tensile test rate of 50 mm/min. According to ISO 180:2000, the impact performance of the test samples was tested by a cantilever arm impact testing machine (model XJU-22, Chengde Testing Machine Co., Ltd., Chengde, China), and the pendulum energy was 5.5 J. The data were recorded for comparative analysis.

### 2.6. Vicat Softening Temperature Test

The Vicat softening temperature of the injection moulded parts made by purchased PA12 pellets, the recycled pellets and the recycled powder were tested separately, and each group was tested with three broken samples obtained by the impact test. According to the test standard ISO 306:1994, the temperature was measured by a thermal deformation Vicat tester (model ZWK1302-A, MTS Industrial Systems CO., LTD., Shanghai, China). The test conditions were as follows: the weight was 50 N, and the heating rate was 120 °C/h.

### 2.7. Scanning Electron Microscopy (SEM)

After the impact test, the fracture surface of the test strip was used to observe morphological patterns made from different raw materials and different printing directions. The surface of the sample was covered with a layer of gold powder, and the fracture surface was imaged at 15 kV using a field emission scanning electron microscope. The damage mode of different print direction test samples was observed.

### 2.8. X-ray Analysis (XRD)

The wide-angle X-ray diffraction (XRD) spectrum of the PA12 material was determined in reflection mode on a diffractometer using a Ni-filtered Cu Kα radiation source λ = 1.5406 Å, a scan rate of 3°/min, a voltage of 40 kV, and a current of 30 mA over the range 2θ: 10–35°. Scanning was performed on samples printed from recycled powder and nylon pellets.

## 3. Results and Discussion

### 3.1. Comparison of Performance of Recycled PA12 Powder and PA12 Pellets

This experiment compared the flowability of purchased PA12 pellets, pellets prepared with recycled powder, and recycled powder. The tensile, flexural and impact properties and heat resistance of the injection molded test samples were tested, and the strips were tested for the Vicat softening point. The results are shown in Table 1.

As shown in Table 1, compared with the fresh material, the MFR of the recycled powder decreased by 77.19%, and the MFR of the recycled powder pellet decreased by 64.96%. The melt fluidity of the recycled powder is significantly lower than that of the fresh material, and the fluidity of the powder is slightly improved after extrusion granulation. In the extrusion granulation process, in the twin-screw extruder, due to the high level of friction between the powder and the screw and the inner wall of the machine, the actual processing temperature is much higher than the set temperature, the presence of oxygen leads to oxidative degradation. Under the action of high temperature and shearing force, the polymer radicals are decomposed to initiate an automatic oxidation cycle. On the one hand, the chain growth reaction occurs, and on the other hand, the carbonyl compound is decomposed. The main effect of these reactions is chain scission, which leads to a decrease in molecular weight and an increase in melt mass flow rate.

The overall reduction in the fluidity of the recycled powder is the result of SLS being carried out with a very low oxygen content. To prevent the warpage deformation of the SLS part, the powder material needs to be pre-heated. The preheating temperature is close to the melting temperature of the PA12, and the condensation reaction of the PA12 occurs under high temperature and without oxygen; the reaction equation is:

H[HN(CH_2_)_11_CO]_n_OH + H[HN(CH_2_)_11_CO]_m_OH → H[HN(CH_2_)_11_CO]_n_ + _m_OH + H_2_O,

The condensation reaction leads to a multiplication of the molecular weight of PA12, and the larger the molecular weight, the more complicated the entanglement of the polymer chain and the more the entanglement point, thus resulting in poor fluidity and a lower melt mass flow rate.

Compared with the tensile strength and the impact strength of the test sample injection molded from fresh PA12 pellets, the corresponding performance of the test sample injection molded from recycled PA12 powder was reduced by 20.23% and 23.65%, and the test sample injection molded from pellet made from recycled powder was reduced by 7.39% and 19.59%. The performance of the recycled powder was not completely consistent. When selective laser sintering was performing, the degree of aging of the powder at different positions was different, and the performance was relatively uniform after extrusion granulation, so the strength was improved, but the rework will reduce the molecular weight, and the performance was still worse than that of the fresh pellet. At the same time, the crystallinity of the powder material and the spherulite size increase, so the impact strength decreases.

Compared to the test sample molded from fresh PA12 pellets, the flexural modulus of the test sample injection molded from recycled PA12 powder and the test sample injection molded from pellet made from recycled powder did not change much, only a slight increase. The Vicat softening temperature of the recycled powder was slightly higher than that of the fresh pellet, and the Vicat temperature increased by approximately 3 °C after powder granulation. This was because the powder material had undergone an ageing process, and the condensation of PA12 molecules occurred. The molecular chain becomes longer, and the molecular weight increases, resulting in an increase in the inter-molecular force and the inter-chain entanglement, which ultimately leads to an increase in the heat resistance of the PA12 powder and the Vicat softening temperature.

### 3.2. The Effect of Printing Speed on the Mechanical Properties of Printed Samples

In this experiment, the recycled PA12 powder was granulated, drawn into a cylindrical filament of 1.75 (±0.05) mm, and finally printed by an FDM printer. The FDM printer sets the print speed gradient. If the printing speed is too fast, serious warpage may occur. Therefore, the printing speed was set to 30, 40, and 50 mm/s. After completing the printing, the mechanical properties of the test sample were tested. During the printing process, the product formation process, the surface warpage phenomenon, and the contour profile were observed, and the adhesion between the layers can be felt by hand touching. In the process of printing the product, if there is serious warpage, it needs to be reprinted, and the sample without warping is used to measure the mechanical properties. The performance is shown in Table 2.

As the printing speed increases, the tensile strength gradually decreases, reflecting the law that the printing speed is inversely proportional to the tensile strength of FDM printed test samples. The slower the printing speed, the more uniform the nozzle spins, the smaller the gap between the filaments, the larger the contact surface, and the better the compactness of the print. As the printing speed increases, the quality of the print decreases slightly, and the density and tensile strength also tend to decrease.

During the printing process, when the polymer is in a molten state, the macromolecular chain arrangement is disordered. During the crystallization process, a portion of the macromolecular chain forms an ordered arrangement. When the printing speed is low, the gap between the molecular chains is small, and the degree of crystallization is higher. Fewer voids lead to more difficulty in releasing the stress, and the larger the volume shrinkage, the more likely that warpage deformation occurs. However, a higher printing speed leads to a shorter printing time and less pronounced warpage of the printed test sample, but the interlayer adhesion is deteriorated, so the tensile strength is low.

The change in printing speed has little effect on the flexural modulus of the printed test sample. In the FDM process, one layer is deposited first, and then another layer is deposited, which is stacked layer by layer. Therefore, the adhesion between layers has a great influence on the performance of the printed product. When the printing speed is 30 mm/s, the cooling time of each layer is longer, the adhesion of the extruded semi-solidified material to the next layer is worse, so the flexural modulus is lower.

The printing orientation used in this test is *x*-direction printing. The specific details are covered in Chapter 2 (2.4). The impact strength is particularly affected by the orientation of the printing test sample layers. The orientation between the layers in the *x*-direction is perpendicular to the orientation of the impact pendulum force. As the printing speed increases, the interlayer adhesion of the printed test sample deteriorates, and the impact strength gradually increases.

Based on the above analysis, the printing speed has a certain influence on the mechanical properties. The PA12 filament has better mechanical properties at a printing speed of 40 mm/s.

### 3.3. Comparison of Printing Performance between Recycled PA12 Filament and Fresh PA12 Filament

**Figure 2:**
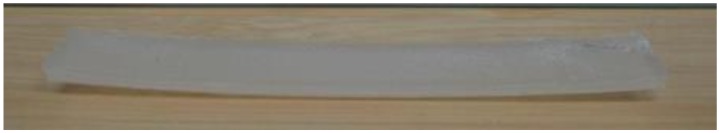
Test sample printed by fresh PA12.

**Figure 3:**
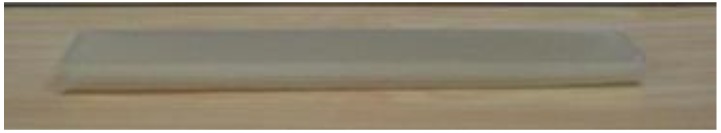
Test sample printed by recycled PA12.

Fused deposition modelling is an unstable process and the diameter of filaments extruded by nozzles is not consistent. The diameter of polybutyrate-adipate-terephthalate–polymer (PBAT) filament measured by Sarat Singamneni et al. [23] shows that the measured value decreases continuously. Moreover, in the printing process, due to the large shrinkage rate of pure PA12 material, warp deformation easily occurs. As shown in Figure 2, the printed test sample is not firmly bonded to the bottom plate and even lift from the hot bed during printing. It is difficult to print a complete test sample. However, the printing performance of recycled PA12 powder is obviously better than that of the new materials. As shown in Figure 3, although the phenomenon of warping deformation sometimes occurs, the degree of warpage is smaller. The performance of the PA12 recycled powder after selective laser sintering is greatly changed, the shrinkage rate and shrinkage deformation phenomenon of the PA12 material are lowered, and the printing performance is superior to that of the untreated pure PA12 material.

**Figure 4:**
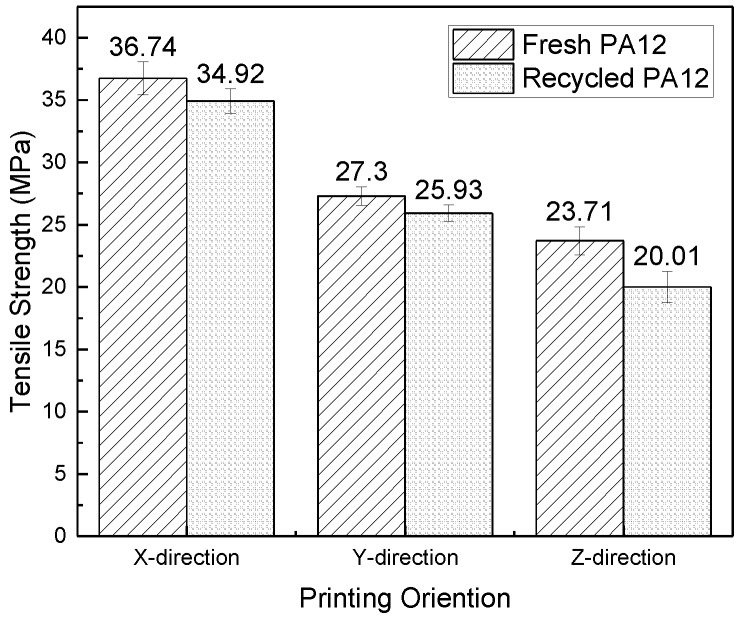
Tensile strength of test samples in different printing orientations.

**Figure 5:**
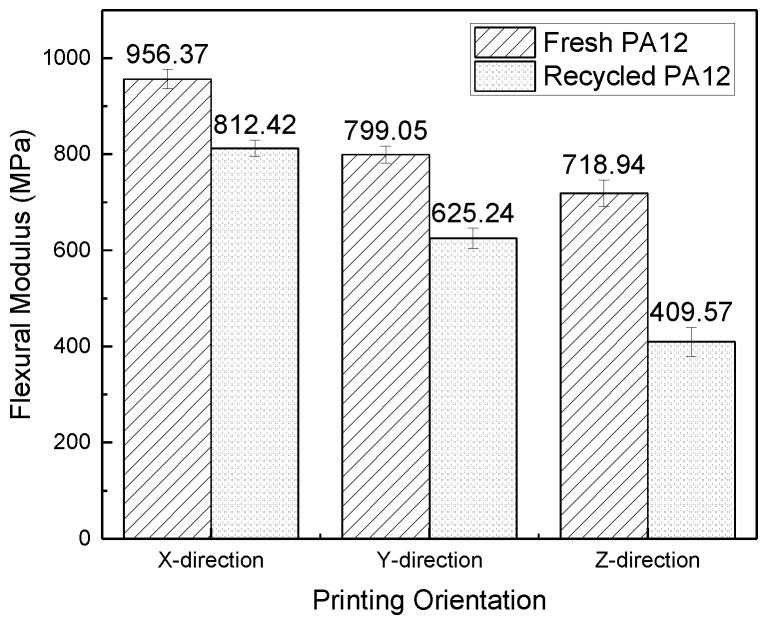
Flexural modulus of test samples in different printing orientations.

**Figure 6:**
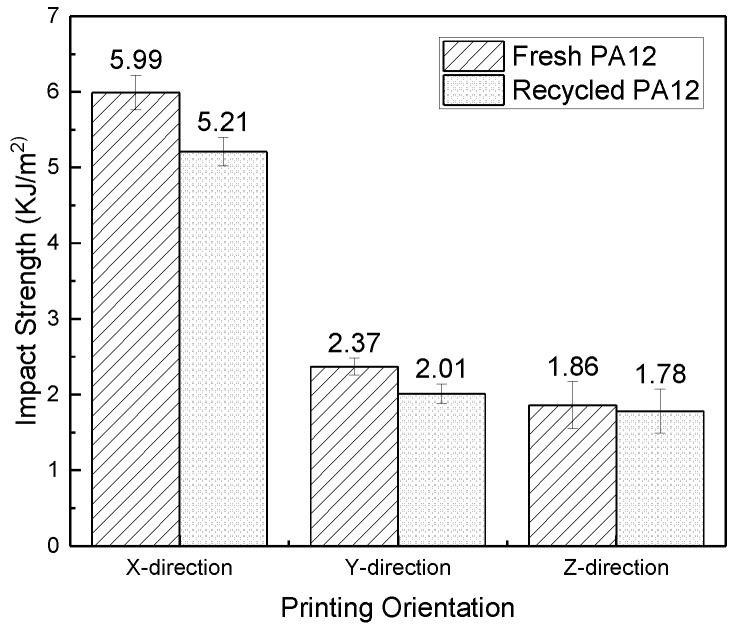
Impact strength of test samples in different printing orientations.

Test samples with standard shapes and sizes, no warping deformation, and close bond were collected, and then their tensile, flexural and impact properties were tested according to the method in Section 2.5. The test results are shown in Figure 4, Figure 5 and Figure 6.

As shown in Figure 4, Figure 5, and Figure 6, the tensile strength, flexural modulus, and impact strength of the test sample printed in the *x*-direction are the best, the *y*-direction is the second, and the *z*-direction is the worst. The tensile strength, flexural modulus, and impact strength of the test sample of the *y*-direction and *z*-direction printed from filaments made of recycled powder reached 74% and 57%, 77% and 50%, and 39% and 34% of the *x*-direction print test samples, respectively. For different printing directions, the size of the bonded area (as shown in Figure 1) is significantly different, and the larger the bonded area, the better the bonding. The bonding area of the *z*-direction print test sample is the smallest. When the tensile test is performed, the force acts on the two layers, and the adhesion between the layers resists deformation during the flexural test. In the case of impact fracture, the *x*-direction test sample is prone to inter-layer fracture, while the *x*-direction and *y*-direction test samples exhibit cross-layer fracture, so the mechanical properties in the *x*-direction and the *y*-direction are better.

The tensile strength and impact strength of the test samples printed from filament made of fresh PA12 pellets were slightly higher than the test samples made of the recycled powder. For the flexural performance, the *x*-direction test sample, *y*-direction test sample, and *z*-direction test sample printed from filaments made of recycled powder reached 85%, 78%, and 57% of the performance of the corresponding test samples made of fresh pellets, respectively. Because the recycled material has been processed and sintered many times, various properties have changed for various reasons, such as the mechanical shearing during processing. Although the mechanical properties of the recycled materials and fresh materials are not much different, the mechanical properties of the recycled materials are slightly lower than those of the fresh materials.

### 3.4. Scanning Electron Microscopy

**Figure 7:**
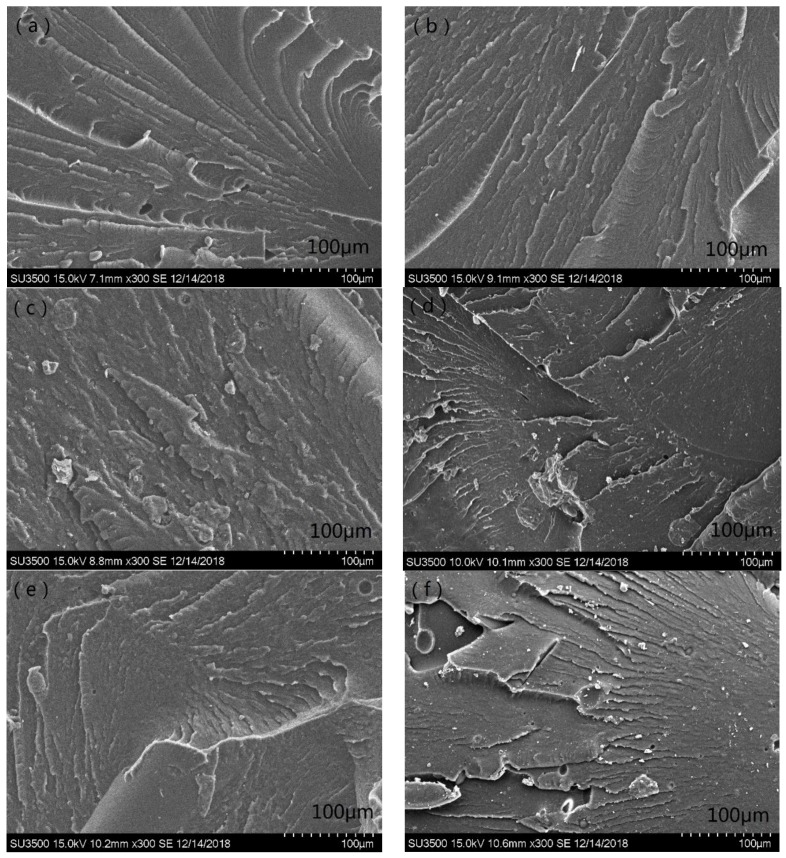
X-direction (**a**), *y*-direction (**c**), and *z*-direction (**e**) test samples printed from filament made of fresh pellets; *x*-direction (**b**), *y*-direction (**d**), and *z*-direction (**f**) test samples printed from filament made of recycled powder.

Figure 7 shows SEM images of the impact fracture interfaces of *x*-direction, *y*-direction, and *z*-direction samples printed from filaments made of fresh PA12 pellets and recycled PA12 powder.

The fracture morphology is substantially similar to a line-like texture left by layer accumulation. During the printing process, each thread is bonded to each other to form a layer and rapidly solidifies after deposition. Subsequent layers, which are still hot, will fuse with the previous layer, and the process continues until all layers of the part are completed. However, there is no obvious delamination, and it can be seen that the printed test samples still have a relatively uniform material structure, which is advantageous for the mechanical properties of the product. At the same time, some pits and small holes were observed, which indicates that the material is not completely dense and does not reach the desired molten state; thus, the printing temperature still needs to be improved.

### 3.5. XRD Analysis

**Figure 8:**
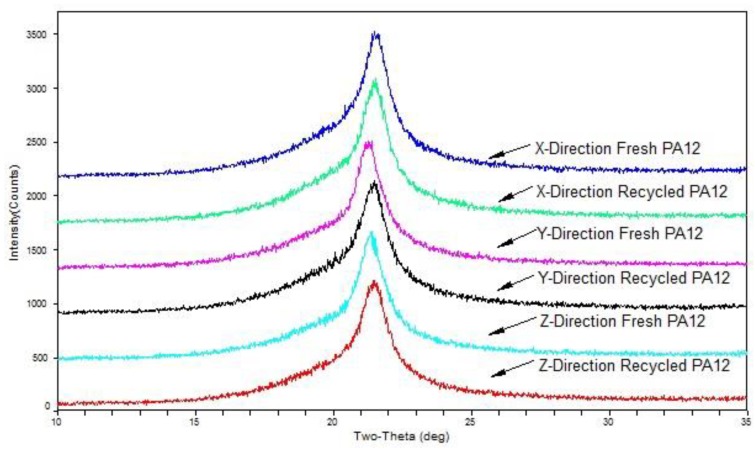
XRD pattern of different build orientations printed from filaments made of recycled PA12 powder and fresh PA12 pellets.

The PA12 may be arranged in a parallel or anti-parallel orientation to form α- and γ-type crystals, respectively. When all polymer chains are oriented in an anti-parallel manner, γ-type crystals are produced, in which case the highest crystallinity is obtained. It has been demonstrated that under most processing conditions, the liquid phase will crystallize into the γ-form, which is the most commonly crystalline form in PA12. However, certain conditions lead to the α-form. These have been reported as solution casting (under atmospheric pressure or reduced pressure, below 90 °C), drawing just below the melting point, and high-pressure crystallization [24,25,26]. All samples used in this experiment were treated by air-cooling to room temperature after printing and then subjected to X-ray diffraction analysis. Figure 8 shows an XRD pattern of test samples of different build orientations printed from filaments made of fresh PA12 pellets and recycled PA12 powder.

The crystallinity of the *x*-direction, *y*-direction, and *z*-direction test samples printed from filaments made of PA12 pellets were 21.04%, 27.50%, and 32.04%, respectively, and the crystallinity of the printed samples of the recycled PA12 powder were 20.07%, 27.30%, and 28.35%. The crystallinity of the test sample made of the recycled powder decreased, but the decrease was not large. Whether prepared from recycled PA12 powder or fresh PA12 pellets, the crystallinity of the test sample printed in the z-direction was the highest, the *y*-direction was the second, and the *x*-direction was the lowest. Different printing directions corresponded to sections of different sizes because the printing speed was the same; both were 40 mm/s, resulting in different cooling times of the filaments in each layer of molten state, and the cooling time in the *z*-direction was the longest. The high cooling rate during polymer processing can result in the formation of unstable γ-crystals, which contribute to the improvement of crystallinity.

## 4. Conclusions

This study aimed to recycle PA12 powder after SLS and develop a filament that can be used for FDM. In this study, PA12 powder after SLS was recycled and pelletized, and the injection was then molded. Compared with the fresh PA12 pellet injection molding, the tensile strength decreased by 7%, the flexural modulus barely changed, the impact strength decreased by 20%, and the overall mechanical properties declined slightly with the recycled powder. The melt fluidity of the recycled powder decreased by approximately 65%, but it was still able to be drawn and printed. The effects of different printing speeds on the mechanical properties of the test samples were tested. Finally, 40 mm/s demonstrated better mechanical properties and was selected as the printing speed for subsequent research. In the samples printed from filaments made of fresh PA12 pellets or recycled PA12 powder, it was found that the mechanical properties of the test samples printed in the *x*-direction were the best, the *y*-direction was second, and the *z*-direction was the worst, and the mechanical properties of the test samples printed by the new pellets were slightly better. Scanning electron microscopy and X-ray diffraction experiments were carried out, and it was found that the test samples made of fresh PA12 pellets and the recycled PA12 powder had similar microscopic morphology and good compactness, but further improvement was needed. The crystallinity of the two test samples was not much different. The crystallinity of the test sample prepared from filaments made with fresh PA12 pellets was slightly higher, and the crystallinity levels were ranked as *x*-direction < *y*-direction < *z*-direction.

The recycled PA12 powder can be used in FDM, and the performance of the recycled PA12 is not much different from that of the fresh PA12 pellets. Moreover, the cost of recycled powder is low, and if it can be successfully applied in the future, it not only solves the problem of SLS waste but also reduces the cost of molten deposition molding, which is beneficial to the entire 3D printing industry. Future research will focus on the preparation of composite filaments for recycled powders and the improvement of other performance tests to produce a filament with low shrinkage and excellent mechanical properties.

## Figures and Tables

**Table 1:** Performance comparison of Polyamide 12 (PA12) powder and fresh material.

Performance Testing	MFR (g/10 min)	Tensile Strength (MPa)	Flexural Modulus (MPa)	Impact Strength (kJ/m^2^)	Vicat Softening Temperature (°C)
Recycled powder	6.99	37.35	815	3.39	138.65
Pellet made from recycled powder	10.74	43.36	807	3.57	141.75
Fresh pellets	30.65	46.82	793	4.44	138.2

**Table 2:** Effect of printing speed on mechanical properties.

Printing Speed	Tensile Strength (MPa)	Flexural Modulus (MPa)	Impact Strength (kJ/m^2^)	Density (g/cm^3^)
30 mm/s	38.4	800	4.41	0.98
40 mm/s	34.92	812	5.21	0.97
50 mm/s	33.27	853	5.42	0.95
